# Wysokie Odejście Moczowodu Przyczyną Skrajnego Wodonercza u 5-letniego Dziecka z Nerką Podkowiastą

**DOI:** 10.34763/devperiodmed.20182204.371375

**Published:** 2019-01-14

**Authors:** Grażyna Krzemień, Agnieszka Turczyn, Małgorzata Pańczyk-Tomaszewska, Przemysław Bombiński, Agnieszka Szmigielska

**Affiliations:** 1 Katedra i Klinika Pediatrii i Nefrologii, Warszawski Uniwersytet Medyczny, Warszawski Polska; 2 Zakład Radiologii Pediatrycznej, Warszawski Uniwersytet Medyczny, Warszawski Polska

**Keywords:** nerka podkowiasta, ektopia nerek, złączenie nerek, wodonercze, plastyka połączenia miedniczkowo-moczowodowego, dzieci, horseshoe kidney, renal ectopia, renal fusion, hydronephrosis, pyeloplasty, children

## Abstract

Nerka podkowiasta rozpoznawana jest z częstością 1:400-1:800 urodzeń. U 44-46% dzieci współistnieje z innymi anomaliami rozwojowymi układu moczowego. Najczęściej rozpoznawane jest wodonercze, odpływ pęcherzowo-moczowodowy i zdwojenie moczowodu. Pięcioletni chłopiec został przyjęty do Kliniki Nefrologii z powodu bólów brzucha i wymiotów. W rutynowym badaniu USG jamy brzusznej wykonanym w wieku 9 miesięcy u chłopca rozpoznano nerkę podkowiastą. Kontrolne badania USG wykazywały niewielkie poszerzenie miedniczki i kielichów w nerce lewej. W badaniu USG jamy brzusznej wykonanym przy przyjęciu do szpitala opisano nerkę podkowiastą ze skrajnym wodonerczem w nerce lewej. W badaniu dopplerowskim uwidoczniono 2 tętnice nerkowe po stronie lewej, bez cech ucisku na moczowód nerki lewej. Scyntygrafia dynamiczna nerek, z użyciem 99mTc-EC, wykazała w nerce podkowiastej wodonercze lewostronne z wysokim udziałem nerki w oczyszczaniu (55% ERPF), wydłużonym czasem tranzytu miąższowego i objawami częściowego utrudnienia wydalania na poziomie ujścia miedniczkowo-moczowodowego. W urografii rezonansu magnetycznego opisano powiększoną nerkę lewą do 105 mm, z odcinkowo zwężoną warstwą miąższową, miedniczka w wymiarze przedniotylnym 39 mm, kielichy poszerzone do 26-32 mm, wysokie odejście moczowodu z miedniczki, nerka prawa prawidłowa. Celem ułatwienia odpływu moczu z wodonerczowo zmienionej nerki lewej założono czasowy drenaż wewnętrzy – cewnik typu double-J. W planie plastyka miedniczkowo-moczowodowa.

**Wnioski:**

U dziecka z nerką podkowiastą i niewielkiego stopnia poszerzeniem układu kielichowomiedniczkowego w okresie niemowlęcym, konieczna jest długofalowa obserwacja ze względu na możliwość nasilenia wodonercza w późniejszych latach życia.

## Abstract (en)

The incidence of horseshoe kidney is 1 per 400-800 live births. From 44-52% of the patients with horseshoe kidney have other coexisting abnormalities of the urinary tract, such as hydronephrosis, vesicoureteral reflux and a duplex collecting system. Our patient, a 5-year old boy, was admitted to a pediatric nephrology department because of abdominal pain and vomiting. He had ultrasonography of the abdomen performed for the first time at the age of 9-months and horseshoe kidney was shown. In a control ultrasonography, a mild dilatation of the pyelocalyceal system in the left kidney was described. On the day of admission, an abdominal ultrasound confirmed horseshoe kidney with large left hydronephrosis. Power Doppler ultrasonography showed two renal arteries to the left kidney and no arterial compression on the ureter. Dynamic scintigraphy (99mTc-EC) revealed left-sided hydronephrosis with high isotope intake up to 55% ERPF, a prolonged time of tissue perfusion and signs of subpelvic junction obstruction. Magnetic resonance urography presented an enlarged left kidney, with a diameter of up to 105 mm, a narrow renal cortex, a dilated renal pelvis up to 39 mm in diameter, dilated calyces up to 26-32 mm, and the high insertion of the ureter from the pelvis. The right kidney was normal. To facilitate drainage from the dilated collecting system of the left kidney, a double-J catheter was inserted. Pyeloplasty is planned as the next step of treatment.

**Conclusion:**

In a child with horseshoe kidney and a mild dilatation of the collecting system detected in infancy, long-term follow up is necessary, because of the increased risk of significant hydronephrosis in the future.

## Wstęp

Nerka podkowiasta jest najczęstszą postacią wady związanej z ektopią i zrośnięciem nerek. Rozpoznawana jest u 90% pacjentów ze zrośnięciem nerek, u pozostałych 10% opisywana jest nerka esowata, nerka w kształcie litery L lub nerka plackowata [1]. Nerka podkowiasta występuje z częstością 1:400-1:800 urodzeń [2, 3]. Wada powstaje w pierwszych 4-8 tygodniach embriogenezy [1, 4, 5]. Z powodu zrośnięcia nerek i zaburzenia migracji ku górze, nerka podkowiasta jest nieprawidłowo zrotowana i położona nisko [4, 5]. U 90-95% pacjentów nerki połączone są dolnymi biegunami [1, 2, 5]. Miejsce połączenia obu biegunów tworzy tak zwaną cieśń nerki, która w większości przypadków jest zbudowana z prawidłowej tkanki nerkowej, rzadziej z tkanki dysplastycznej lub włóknistej [5]. W nerce podkowiastej stwierdza się różne warianty unaczynienia tętniczego i żylnego. Sposób unaczynienia nerki może mieć istotne znaczenie przy zabiegach chirurgicznych [1, 2, 4, 5].

U 44-46% dzieci nerka podkowiasta współistnieje z innymi wadami wrodzonymi układu moczowego [6, 7]. Najczęściej rozpoznawane jest wodonercze, odpływ pęcherzowo-moczowodowy (OPM) i zdwojenie moczowodu [1, 2, 6]. Nerce podkowiastej mogą towarzyszyć anomalie rozwojowe układu kostnego, pokarmowego, sercowo-naczyniowego, oddechowego, rozrodczego i nerwowego. Wada rozpoznawana jest w różnych zespołach genetycznych, najczęściej w zespole Turnera, Edwardsa i Downa [4, 5, 6, 8]. U pacjentów z nerką podkowiastą stwierdzono częstsze zachorowania na nowotwory nerki [2, 4]. Ryzyko rozwoju guza Wilmsa jest 1,7 do 7,9 razy większe niż w populacji ogólnej [1].

Celem pracy było przedstawienie przypadku późnego wystąpienia wodonercza u chłopca z nerką podkowiastą.

## Opis przypadku

Pięcioletni chłopiec został przyjęty do Kliniki Nefrologii z powodu utrzymujących się od około 2 tygodni bólów brzucha i wymiotów. Dziecko urodzone z ciąży III porodu III rozwiązanego cięciem cesarskim (stan po cięciu cesarskim) w 39 tygodniu, z masą ciała 3450g, ocenione na 10 pkt Apgar. Przebieg ciąży i prenatalne badania ultrasonograficzne (USG) były prawidłowe. W rutynowym badaniu USG jamy brzusznej wykonanym w wieku 9 miesięcy u chłopca rozpoznano nerkę podkowiastą i niewielkiego stopnia poszerzenie układu kielichowomiedniczkowego (UKM) w nerce lewej - wymiar przednio-tylny (ap) miedniczki 6mm, kielichy poszerzone do 3,5mm. Scyntygrafia statyczna nerek z użyciem znacznika 99mTc-DMSA wykazała nerkę podkowiastą z częściową ektopią nerki prawej, udział nerki lewej w oczyszczaniu wynosił 51% ERPF, nerki prawej 49%. Cystouretrografia mikcyjna była prawidłowa. Chłopiec pozostawał pod opieką poradni nefrologicznej. Kontrolne badania USG wykazywały niewielkie poszerzenie miedniczki i kielichów w nerce lewej. Wielokrotnie wykonywane badania moczu były prawidłowe. W badaniu USG wykonanym 8 miesięcy przed przyjęciem do szpitala stwierdzono w nerce lewej poszerzenie miedniczki w wymiarze ap do 5mm i kielichów do 4 mm. Przy przyjęciu do szpitala stan ogólny chłopca był dobry, w badaniu przedmiotowym stwierdzono: brzuch miękki, bolesność uciskowa w lewej okolicy podżebrowej, perystaltyka prawidłowa, objawy otrzewnowe ujemne, ropna wydzielina pod napletkiem, ciśnienie tętnicze 99/71 mmHg. Badania laboratoryjne wykazały: stężenie mocznika w surowicy 45 mg/dl (norma 15,0-36,4 mg/dl), kreatyniny 0,6 mg/dl (norma 0,2-0,7 mg/dl), cystatyny C 1,05 mg/l (norma 0.51-1,05 mg/l), kalkulowany klirens kreatyniny (GFR) obliczony z wzoru Schwartza wynosił 78 ml/min/1,73 m^2^ (norma 133±27 ml/min/1,73 m^2^), morfologia, CRP, badanie ogólne i posiew moczu prawidłowe. W badaniu USG jamy brzusznej opisano nerkę podkowiastą ze skrajnym wodonerczem w nerce lewej − wymiar ap miedniczki 40mm, kielichy poszerzone do 23 mm, wysokie odejście moczowodu z miedniczki (ryc. 1); nerka prawa i pęcherz moczowy prawidłowe. W badaniu dopplerowskim uwidoczniono 2 tętnice nerkowe po stronie lewej, bez cech ucisku/krzyżowania okolicy podmiedniczkowej moczowodu lewego. Scyntygrafia dynamiczna nerek, z użyciem znacznika 99mTc-EC, wykazała nerkę podkowiastą z częściową ektopią nerki prawej; nerka lewa zmieniona wodonerczowo, ze zwężoną warstwą miąższową, wydłużonym czasem tranzytu miąższowego i objawami częściowego utrudnienia wydalania na poziomie ujścia miedniczkowo-moczowodowego, obraz nerki prawej prawidłowy; udział nerki lewej w oczyszczaniu wynosił 55% ERPF, nerki prawej 44% (ryc. 2). W urografii rezonansu magnetycznego opisano nerkę podkowiastą, nerka lewa powiększona do 105mm, z odcinkowo zwężoną warstwą miąższową do 6 mm i znacznego stopnia poszerzeniem UKM - miedniczka skierowana ku przodowi, poszerzona w wymiarze ap do 39 mm, kielichy poszerzone do 26-32 mm, wysokie odejście moczowodu w połowie miedniczki (ryc. 3), nerka lewa długości 88 mm, prawidłowa. Celem ułatwienia odpływu moczu z wodonerczowo zmienionej nerki lewej, założono czasowy drenaż wewnętrzny – cewnik typu double-J. W planie plastyka miedniczkowo-moczowodowa.

**Ryc. 1:**
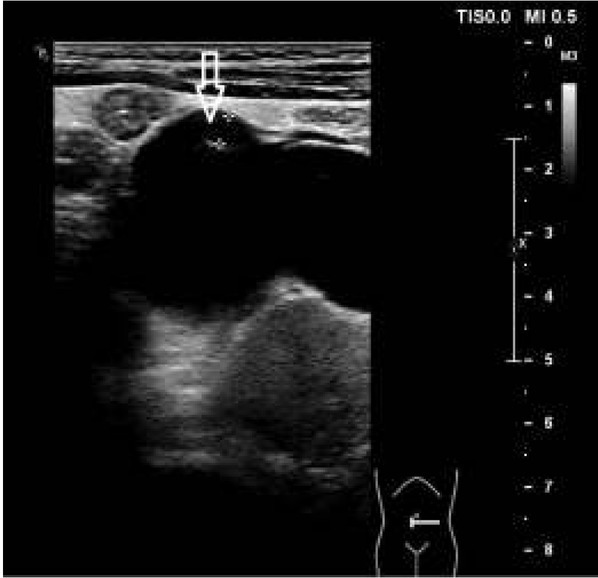
Badanie USG – widoczne wodonercze lewego układu i wysokie odejście lewego moczowodu. Fig. 1. Ultrasonography images of left collecting system hydronephrosis and high insertion of the left ureter.

**Ryc. 2:**
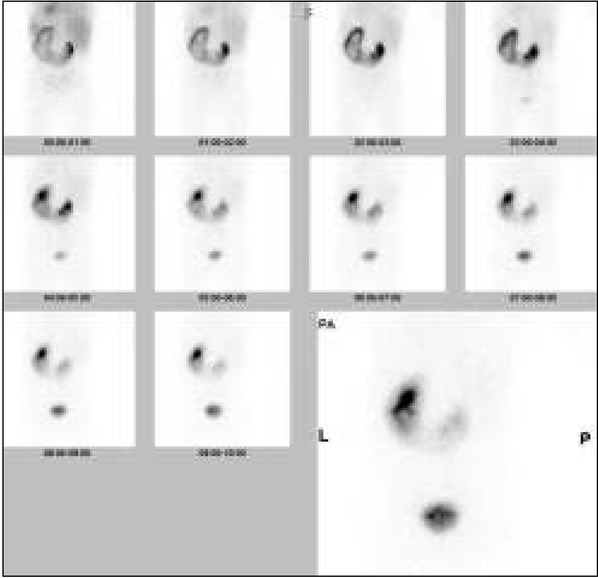
Scyntygrafia dynamiczna nerek z użyciem 99mTc-EC nerki podkowiastej z wodonerczem lewostronnym, z objawami częściowo utrudnionego wydalania na poziomie ujścia miedniczkowo-moczowodowego. Fig. 2. Dynamic scintigraphy with 99mTc-EC of horseshoe kidney with hydronephrosis of the left-sided pelvicalyceal system and partial obstruction at the level of the uretropelvic junction.

**Ryc. 3:**
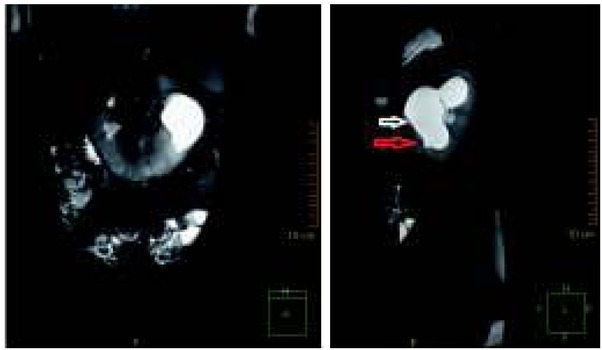
T2-zależne rekonstrukcje czołowa i strzałkowa urografii rezonansu magnetycznego nerki podkowiastej, z wodonerczem lewego układu i wysokim odejściem lewego moczowodu; biała strzałka – odejście moczowodu, czerwona strzałka – moczowód. Fig. 3. Coronal and sagittal T2-weighted magnetic resonance urography images of horseshoe kidney, with hydronephrosis of the left collecting system and high insertion of the ureter.

## Omówienie

U około 35% dzieci z nerką podkowiastą rozpoznawane jest wodonercze [2, 6]. Najczęściej powodem wodonercza są zaburzenia w odpływie moczu spowodowane nieprawidłowym położeniem miedniczki i wysokim odejściem moczowodu z miedniczki lub uciskiem moczowodu przez dodatkowe naczynie lub cieśń nerki podkowiastej [2, 4, 7]. U około 10% dzieci z wysokim OPM może dojść do rozwoju wtórnego wodonercza z powodu zagięcia wydłużonego i pofałdowanego moczowodu [9]. Rzadko przyczyną wodonercza, u pacjentów z nerką podkowiastą, jest śródścienne zwężenie moczowodu w okolicy połączenia miedniczkowo-moczowodowego [10, 12].

W większości przypadków nerka podkowiasta nie daje żadnych objawów klinicznych [3, 4, 6]. Najczęściej rozpoznawana jest w przypadkowo wykonanym badaniu USG jamy brzusznej (29,3%) lub prenatalnie (12,2%). U pozostałych dzieci wskazaniem do wykonania badania USG są bóle brzucha (19,5%), zaburzenia mikcji (17,1%), zakażenie układu moczowego (ZUM) (9,8%) i krwiomocz (7,3%) [6]. U około 5% chorych może być wyczuwalny guz w brzuchu [4]. Wystąpienie objawów klinicznych ma związek z obecnością dodatkowych powikłań w układzie moczowym – wodonercza, ZUM lub kamicy nerkowej [4, 6]. Zakażenie rozpoznawane jest u 27-41% pacjentów, kamica nerkowa u 3% dzieci i u 36% dorosłych z nerką podkowiastą [2, 12]. U opisanego chłopca USG prenatalne było prawidłowe. Nerkę podkowiastą rozpoznano w rutynowym badaniu jamy brzusznej w wieku 9 miesięcy. Wykonywane w kolejnych latach badania USG wykazywały niewielkie poszerzenie miedniczki i kielichów w nerce lewej. Przez 5 lat obserwacji w poradni nefrologicznej dziecko nie demonstrowało żadnych objawów klinicznych związanych z obecnością wady układu moczowego. W wieku 5 lat z powodu bólów brzucha i wymiotów wykonano badanie USG, które wykazało skrajne wodonercze w nerce lewej.

U dzieci z nerką podkowiastą i wodonerczem badania obrazujące układ moczowy obejmują USG, scyntygrafię dynamiczną nerek i cystouretrografię mikcyjną. U części chorych przed planowanym leczeniem operacyjnym konieczne jest wykonanie urografii rezonansu magnetycznego lub urografii tomografii komputerowej. Badania te pozwalają dokładnie zobrazować współistniejącą wadę układu moczowego oraz sposób unaczynienia nerki podkowiastej [1, 4, 13, 14]. Leczenie chirurgiczne jest konieczne u 17-55% pacjentów z nerką podkowiastą [6, 7]. Kwalifikacja do plastyki połączenia miedniczkowo-moczowodowego, relokacji naczynia dodatkowego czy korekcji OPM u dziecka z nerką podkowiastą, odbywa się według takich samych zasad, jak w przypadku nerek prawidłowo położonych. Wskazaniem do leczenia operacyjnego wodonercza są: 1) cechy uszkodzenia nerki w badaniu izotopowym – udział w oczyszczaniu poniżej 40% lub obniżanie w kolejnych badaniach o 5-10% i zablokowany odpływ moczu z nerki; 2) nasilone wodonercze w badaniu USG – wymiar ap miedniczki nerkowej powyżej 20 mm lub wodonercze 3-4 stopnia według klasyfikacji Society for Fetal Urology; 3) objawy kliniczne będące następstwem zastoju moczu – kolka nerkowa, ZUM, kamica nerkowa [11, 15].

U naszego pacjenta powodem wodonercza było wysokie odejście moczowodu z miedniczki. Urografia rezonansu magnetycznego pozwoliła wykluczyć ucisk moczowodu przez dodatkowe naczynie lub cieśń nerki podkowiastej. Wskazaniem do leczenia zabiegowego było skrajne wodonercze ze zwężeniem warstwy miąższowej nerki w badaniu USG i urografii rezonansu magnetycznego, objawy utrudnionego wydalania moczu na poziomie ujścia miedniczkowo-moczowodowego w badaniu izotopowym oraz obserwowane u dziecka objawy kolki nerkowej. Wbrew oczekiwaniom, scyntygrafia nerek wykazała wysoki udział nerki lewej w oczyszczaniu. U dziecka ze skrajnym wodonerczem udział nerki w oczyszczaniu może być zawyżony z powodu przedłużonego zliczania impulsów izotopu, zalegającego w poszerzonym UKM.

## Podsumowanie

U dziecka z nerką podkowiastą i niewielkiego stopnia poszerzeniem układu kielichowo-miedniczkowego w okresie niemowlęcym, konieczna jest długofalowa obserwacja ze względu na możliwość nasilenia wodonercza w późniejszych latach życia.
